# Obsessive-compulsive disorder and related disorders: a comprehensive survey

**DOI:** 10.1186/1744-859X-8-13

**Published:** 2009-05-18

**Authors:** Michele Fornaro, Filippo Gabrielli, Claudio Albano, Stefania Fornaro, Salvatore Rizzato, Chiara Mattei, Paola Solano, Valentina Vinciguerra, Pantaleo Fornaro

**Affiliations:** 1 Dipartimento di Neuroscienze, Oftalmologia e Genetica (DINOG), Sezione di Psichiatria, Università di Genova, Genova, Italy; 2 Dipartimento di Neuroscienze, Oftalmologia e Genetica (DINOG), Sezione di Neurologia, Università di Genova, Genova, Italy; 3 Dipartimento di Neuroscienze, Sezione di Medicina Legale, Università di Pisa, Pisa, Italy; 4 Dipartimento di Psichiatria, Neurobiologia, Farmacologia e Biotecnologie, Sezione di Psichiatria, Università di Pisa, Pisa, Italy

## Abstract

Our aim was to present a comprehensive, updated survey on obsessive-compulsive disorder (OCD) and obsessive-compulsive related disorders (OCRDs) and their clinical management via literature review, critical analysis and synthesis.

Information on OCD and OCRD current nosography, clinical phenomenology and etiology, may lead to a better comprehension of their management. Clinicians should become familiar with the broad spectrum of OCD disorders, since it is a pivotal issue in current clinical psychiatry.

## Introduction

Obsessive-compulsive disorder (OCD) is a common, chronic, anxiety condition that can have disabling effects on both genders throughout the patient's lifespan. OCD can manifest with a wide range of clinical pictures [1].

The disorder is among the most disabling anxiety conditions and counts for more than half of serious anxiety cases [2]. However, no univocal clinical opinion exists about its classification. In fact, although the Diagnostic and Statistical Manual of Mental Disorders, 4th edition – text revision (DSM-IV-TR) [3] classifies OCD as an anxiety disorder, some clinicians conceptualize it as a spectrum of related disorders (OCRDs) sharing the 'anxiety/fear' coupled with 'worry' clinical feature [4,5].

The broad spectrum of OCRDs includes the somatoform disorders (for example, body dysmorphic disorder (BDD) and hypochondriasis), the impulse-control disorders (for example, trichotillomania (TTM), pathological gambling, skin picking and others) and the tic disorders (for example, Tourette's syndrome) but others, including drug-induced and non-psychiatric disorders, could overlap and show similar clinical pictures [6]. The National Comorbidity Survey Replication study reported more than a quarter of evaluated subjects developing obsessions and compulsions at some point in their life and possibly manifesting with a full-threshold OCD, while a higher number of patients will probably suffer from OCRDs [2].

The most common age of onset of OCD is reported to be between 22 and 35, while affected patients spend an average of 17 years before receiving a correct diagnosis and treatment, with most OCD and OCRDs often showing a waxing and waning course, frequently increasing in severity when left untreated [7,8].

Further increasing the burden of OCD is the fact that affected subjects, along with many psychiatric patients, often experience discrimination and stigmatization due to a non-medical perception of the phenomenon. Yet OCD and OCRDs represent relevant medical conditions. Findings provided by recent studies, mainly focusing on the role played by the amygdala and its links to the 'fear circuits' and other structural and functional abnormalities of several corticostriatal pathways, also indicate a relationship between OCD manifestations and its neurobiological basis, suggesting new therapeutic strategies [9].

Treatment of OCD typically involves the use of medications in combination with other modalities (such as cognitive behavioural therapy (CBT), psychoeducation and support groups and so on): first line treatments options include both serotonin reuptake inhibitors (SRIs) medication and CBT [10], but anxiolitics and antipsychotics, among other classes of drugs, are used as well. Finally, the identification of OCD and its appropriate treatment is essential to improve the quality of assistance and to reduce the waste of health care resources through unnecessary medical care.

## Historical background and current nosography

Obsessions thoughts and compulsive urges or actions are part of everyday life. We return to check that we locked a door and switched off the light. We cannot stop thinking about the stressful event scheduled for the next week. We refuse to eat with the spoon that dropped on the floor, even if we know the chance of contamination is remote.

These events are part of the normal feedback and control loop between our thoughts and our actions, and they have an ancestral biological survival value. It is only when obsessive thoughts become frequent or intense, or unavoidable, or when these compulsive rituals become so prominent that they interfere with an individual's functioning, that the diagnosis of OCD is made.

Descriptions of the phenomena of obsessions and compulsions can be found in historical documents over the past several centuries, since OCD has a long history. A passage from the *Malleus Maleficarum*, the 15th century compendium of witchcraft and psychopathology, describes a priest brought to Rome for exorcism:

' [w]hen he passed any church, and genuflected in honour of the Glorious virgin, the devil made him thrust his tongue far out of his mouth when he tried to engage in prayer, [the devil] attacked him more violently' [11].

Those with obsessive thoughts of a blasphemous or sexual nature were thought to be partially possessed by the devil, while 'psychotic' individuals appeared fully possessed. Obsessions and hand-washing rituals resulting from guilt were immortalized in the 17th century by the Shakespeare character Lady Macbeth:

'[...] it is an accustomed action with her, to seem thus washing her hands. I have known her continue with this a quarter of an hour' (Macbeth, V.i.28, describing the time-wasting characteristic of OCD).

With time, the explanation for obsessions and compulsions moved from a religious view to a medical one. Obsessions and compulsions were first described in the psychiatric literature by Esquirol in 1838, and, by the end of the 19th century, they were generally regarded as manifestations of melancholy or depression. By the beginning of the 20th century, the view of obsessive-compulsive phenomena had begun to shift OCD toward a psychological explanation; Janet had already described the successful treatment of compulsive rituals with what would come to be known behavioral techniques [12], and with Freud's publication in 1909 of the psychoanalysis of a case of obsessional neurosis (the Rat Man), obsessive and compulsive actions came to be seen as the results of unconscious conflicts and the isolation of thoughts and actions from their emotional components [13]. Although this shift succeeded in pointing out that actions can be motivated by factors of which the individual is unaware or unable to control, it did little to improve the outcome of patients OCD.

In the 1950s, with the rise of behavioral therapy, the learning theories that had proved to be helpful in the conceptualization and treatment of phobic disorders were applied to OCD symptoms. Although these learning theories are clearly insufficient to account for all OCD (as well as OCRD) symptoms, they did lead to the development in the late 1960s and early 1970s of effective treatments for reducing compulsive rituals. During the 1980s, research focused on the relationship of OCD and neurological problems such as epilepsy [14], memory disorders and Tourette's syndrome [15] while Westphal's early observation of an association between obsessions, tic disorders and epilepsy already presaged recent neurobiological findings in OCD.

**Figure 1:**
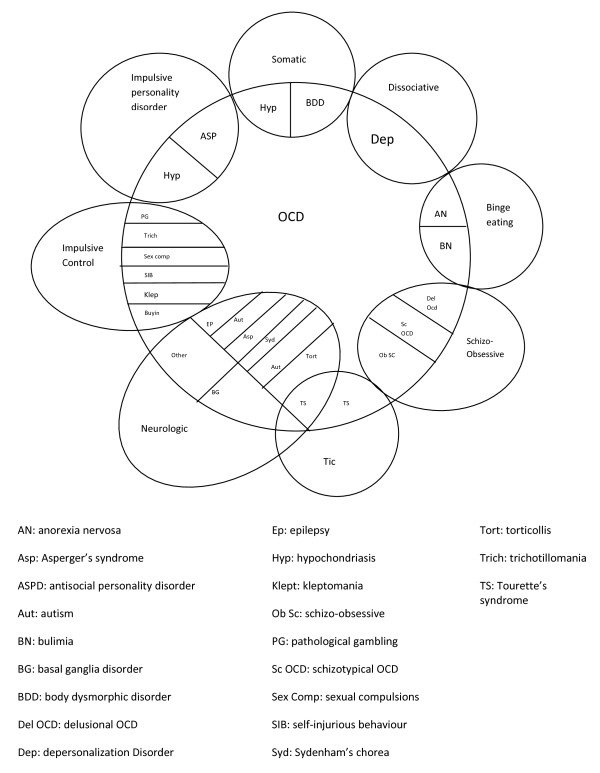
**The current spectrum of obsessive-compulsive disorder (OCD) and related disorders (OCRDs)**. Adapted from Roan WM et al. [17].

OCD and OCRDs may also have common manifestations and, since the 1990s, they have therefore been conceptualized with a broad spectrum of related disorders [16] (Figure 1) [17].

The 1994 DSM-IV operated a split between the phobic/anxious-avoidant and obsessive dimensions, categorized by the previous DSM-III (1980) and its 1987 revised edition (DSM-III-R) with the unitary diagnosis of 'phobic-obsessive disorder' [18]. The current DSM-IV-TR describes OCD as characterized by repetitive thoughts, images, impulses that intrude on a patient unable to stop them [3].

Current nosology underlines the following three major symptom clusters for OCD and OCRDs: a 'somatic' cluster for BDD and hypocondriasis, a 'reward deficiency' cluster for TTM and other impulse control disorders (ICDs), and an 'impulsivity' one for kleptomania, compulsive shopping (CS), pathological gambling (PG), intermittent explosive disorder (IED) and others [19].

While most psychiatrists generally agree on the OCD spectrum including anxious and phobic manifestations, a greater number also focus on the need of a clear-cut definition of anxious and obsessive symptoms, as is anticipated by the research agenda for DSM-V[5]. In fact, anxious phobia differs from OCD. Both phobic and obsessive-compulsive subjects usually avoid feared objects and generally retain awareness their fears and avoidance behaviors are excessive. Phobics are usually more upset about the prospect of actually coming into contact with the thing they fear and do what they can to avoid it, while OCD patients may be more concerned about the time-consuming rituals such contacts will trigger, rather than fear of the contact itself.

## Epidemiology

Obsessive and compulsive symptoms are common and not all of them may be accounted for a full-threshold OCD. Approximately 50% of the general population engage in some ritualized behaviors, while up to 80% experience intrusive, unpleasant or unwanted thoughts [20].

The 1 month prevalence of adult OCD is about 0.6% [21] while the DSM-IV 12 month prevalence ranges from 0.6% to 1%. Regardless, the prevalence of OCD, as well OCRDs, may vary depending on the source of data and the choice of diagnostic instruments. Many OCRDs may co-occur with each other and with OCD. With regard to the somatoform disorders, the estimated prevalence rate of hypochondriasis is 1% to 5% in the general population and 2% to 7% among primary care outpatients. Unfortunately, the prevalence rate of BDD is difficult to estimate given the secrecy of this severe condition [22], but estimates range from 0.7% to 2.3% in the general population and at least from 6% to 15% in cosmetic surgery settings [23]. For OCRDs the prevalence of Tourette's is 0.1%, while the exact lifetime prevalence of TTM is unknown, but rates from 1% to 2% have been reported for cases that satisfy the full threshold diagnostic criteria [24].

There seems to be a bimodal age of onset for OCD. The mean onset has been reported to be 19 years (21% of the cases emerged at age 10), while the mean age for adult OCD occurs between age 22 and 35. In a small number of cases the onset of the disorder occurs at age of 50 or more [2]. Usually, the earlier the age of onset, the worse the course of OCD and OCRDs; by contrast, no specific gender predominance has been reported in large samples epidemiological studies. This latter evidence is in contrast with non-OCD anxiety conditions whose gender ratios usually indicate a prevalence of female cases [25].

While economic, social and cultural effects may play a role in producing different clinical pictures of OCD, biological, immune and genetic factors and family predisposition may also contribute to the pathogenesis of the disorder. For example, streptococcal infection may be associated with an abrupt, exacerbating-remitting early-onset form of OCD, which is termed pediatric autoimmune disorder associated with streptococcus (PANDAS), but little is known about this condition, and in particular about the genesis of this OCRD [26].

OCD's burden may also vary depending on the case in question, on the course of disorder and on the fact it is almost unknown among the general population. As a consequence, many patients do not seek medical care until (originally) milder forms of OCD and OCRDs become more distressful and possibly harder to treat. Furthermore, a large number of obsessive-compulsive conditions may go under-diagnosed: studies have placed the prevalence between 1% and 3% of OCD cases, although the prevalence of clinically recognized OCD is probably much lower [2].

The fact that many individuals do not seek early appropriate treatments may be due to stigma, but also to other factors. Sometimes patients do not realize that they are affected by OCD. In some cases, the 'typically obsessive' features of intrusive, 'ego-dystonic' feelings and thoughts are absent, as in the poor-insight obsessive-compulsive disorder (PI-OCD), complicating the course and severity of the illness [27]. Including PI-OCD and other subtypes extends the range of OCD cases that are reported to afflict approximately 2% to 3% of the world's population; these show varying degrees of severity and chronic course and often also include depressive feelings (80%), major depression (MD) comorbidity (30%) and Tourette's syndrome comorbidity (5%).

Additionally, OCD patients usually present symptoms similar to those of their affected relatives. About 8% of first degree relatives have OCD, while first symptoms occur by their 20s in 75% of the patients; this may happen suddenly or slowly, generally showing an episodic course [28]. Interestingly, the episodic course is sometimes an overlap feature of the illness with MD, but it may also prompt clinicians to explore other affective comorbidities as well [29], and we urge for more vigilance for largely under-recognized entities such as cyclothymic-OCD [30].

For example, a small number of very severe OCD cases may also develop suicidal ideations or behaviors, as the patient could be perceive suicide as the only possibility of escape from their tremendous pain. In such patients, a common neurobiological and genetic basis has been hypothesized to be responsible for depressive suicidal behaviors and for severe ego-dystonic obsessive manifestations. Such a hypothesis has also been supported by the *ex adiuvantibus *findings of similar pharmacotherapeutic strategies being effective in both pathological dimensions.

## Diagnostic criteria

**Figure 2:**
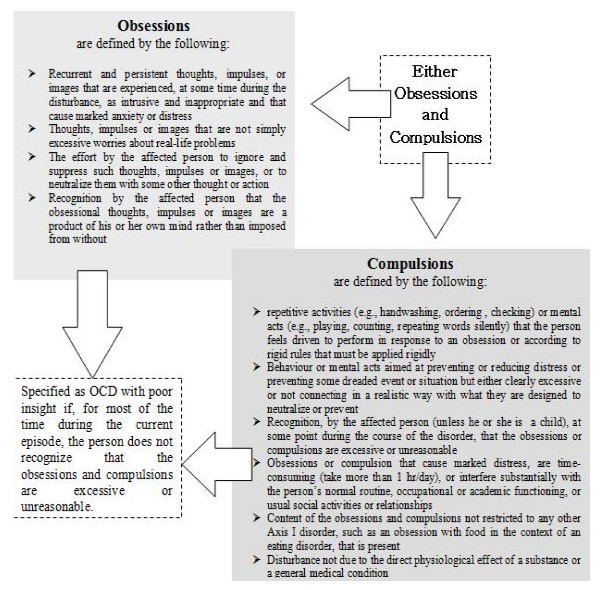
**Diagnostic and Statistical Manual of Mental Disorders, 4th edition – text revision (DSM-IV-TR) criteria for obsessive-compulsive disorder (OCD)**.

The main features of OCD are the obsessions and compulsions. According to DSM-IV-TR, the obsessions and compulsions cause marked distress, are time-consuming (usually taking more than 1 h per day for a month or more) and significantly impair the normal functioning of the subject. If another Axis I disorder is present, it is mandatory that the content of the obsessions or compulsions not be restricted to it (for example, preoccupation with food or weight in eating disorders or guilt ruminations in the presence of a major depressive episode (MDE)). The disturbance should not be due to the direct effects of a substance (for example, drug or medication abuse), or a general medical condition (Figure 2).

Obsessions may also manifest with very heterogeneous clinical pictures (for example, religious scrupulosity, aggressive or intrusive thoughts, inappropriate sexual thoughts, concerns about symmetry and perfectionism, pathological doubt, contamination worries, pathological collecting and hoarding), while compulsions are defined as repetitive behaviors or mental acts (for example, washing, counting, checking, ordering, touching, cleaning, hoarding, conducting mental or physical rituals).

Obsessions are usually unwanted, unavoidable, intrusive, ego-dystonic, occasionally frightening or violent (for example, the impulse to leap before a car, the thought that you may attack your spouse, that the pateint may molest a child) and often impair functioning and quality of life (QoL) [31].

It is remarkable that most OCD patients do criticize their own thoughts and would hate to practice any by choice, yet in most cases they are unable to stop such thoughts or behaviors. Nevertheless, OCD patients can ruminate endlessly ('Did I lock the door?') and most of them develop (new) compulsions to ward off unwanted happenings or to satisfy obsessions (for example, an obsession with dirt leading to hand-washing rituals).

## Differential diagnosis and clinical phenomenology

The word 'obsession' derives from Latin '*obsidēre*', which means 'to take possession', 'to occupy'. In fact, most OCD patients relate to the experience of a 'Middle ages fortress besieged by strong enemies they have to surrender to without any escape possibility'. The Latin word '*compellere*' has the significance of 'to be constrained' and 'to be overpowered': OCD patients are forced to act on compulsions trying to overcome obsessions.

Most OCD patients present both, but occasionally they can manifest only obsessions or compulsions; this is sufficient for OCD diagnosis regardless.

Up to 20% of severe depression cases present obsessive symptoms and treatment maybe identical while schizophrenics often show bizarre rituals they are usually comfortable with, as in schizo-obsessive disorder (SOD) which neurological soft signs (NSS) psychopathology suggests is a severe form of OCRD [32-34].

Differential diagnosis is also a concern due to current OCD and OCRD classification methods. People sometimes wonder if compulsive eating, gambling, shopping or deviant sexual behaviors are forms of OCRD. Usually, these disorders are not classified as OCRDs because some pleasure is obtained by these activities and the person would not, ordinarily, wish to stop them except for the secondary problems they may cause (such as obesity, convictions for driving while intoxicated, gambling and credit card debts and criminal prosecution for sexual deviancy). Nevertheless, few individuals with these compulsive behaviors may respond to drug and behavioral treatments that are effective for OCD [7].

Obsessions usually share an increasing 'anxious tension' before acting the compulsions (both behavioral and mental), followed by a brief sense of relief as they are carried out. This kind of feeling is particularly evident in many OCRDs too, as most eating disorders (EDs) may also be considered. In fact, many bulimia nervosa (BN) patients experience a brief reaction after binge eating while anorexia nervosa (AN) patients take a form of pleasure in being able to keep away from food.

It is important to distinguish between obsessive-compulsive symptoms in the course of EDs and OCD symptoms; a distinction should also be made for 'anxious' feelings experienced in the course of ICDs when the compulsion is carried out.

Occasionally OCD thinking is bizarre, and patients could even exhibit schizotypical personality disorder (SPD) traits, usually being unaware of this (for example, 'My spouse will leave me if I do not catch the elevator').

Since the OCD spectrum phenomenology may be vary heterogeneous, many rating scales and instruments, such as the Yale-Brown Obsessive-Compulsive Scale (Y-BOCS) and others also investigating OCRDs, have been developed to help clinicians make diagnoses and to score symptoms, but clinical interview by a trained psychiatrist should not be discounted in any serious case [35].

## Neurobiology and genetics

There is growing evidence based on several lines of research that OCD and OCRDs involve abnormal metabolism in specific areas of the brain. Neuroimaging findings indicate OCD involves subtle structural and functional abnormalities of the orbito-frontal cortex (OFC), the anterior cingulate cortex (ACC), the caudate nucleus (Cn), the amygdala nuclei (An), the accumbens nucleus (NAc), the cortical thalamic nuclei (Tn) as well the white matter (WM), the hippocampus (HP) and other regions [36].

The OFC is involved with social consciousness regarding proper behavior. Hypoactivity in this area (whether occurring spontaneously or as a result of damage from a perinatal or head injury, temporal lobe epilepsy, infection or brain tumor and other conditions) leads to coarsening of social consciousness and behaviors. This may lead to hypersexuality (paraphilic OCRDs), overeating behavior (EDs and Prader-Willi syndrome (PWS)), personality changes and Tourette's syndrome (frequently presenting with inappropriate use of profanity) or crude jokes (sadomasochistic disorder (SMD)). Overactivity of the OFC may results in excessive social concern, meticulousness and 'nitpicking' habits, fastidiousness and avoidant behaviors and more.

Other brain structures, such as the Cn, filter information coming from the forebrain, representing a sort of hub for many elaborate stimuli. It has been hypothesized that if 'too many' messages regarding worries about 'how things should be done' reach the Cn, they are not filtered properly and spill over into (and flood) consciousness. Increased metabolism of the frontal part of the brain is concerned with order and social proprierty. The Cn, along with other striatal structures, is also involved in regular repetitive behaviors (rituals): the anterior caudatus putamen (aCPu), the ACC directly leading to the shell of the NAc, the pallidus internus and the thalamus may play a specific role in impulsive-repetitive psychic manifestations of OCD and OCRDs (for example, the verbal Tourette's symptoms).

Additionally, the dysregulation of the posterior caudatus putamen (pCPu) and the dorsolateral-prefrontal cortex (DL-PFC), the pallidus internus (PI) and the thalamus may account for the neurological symptoms such as tics, Tourette's motor abnormalities and other OCD spectrum motor issues [32].

Both the striatal and the frontal brain areas are richly supplied with serotonergic neurons. It is not surprising that most OCD and OCRD drugs act as modulators for the serotonergic transmission in the central nervous system (CNS). However, even though 5-hydroxytryptamine (5-HT) is a core neurotransmitter involved in OCD and OCRD manifestations, this knowledge tells us little about the ultimate causes or triggers of this psychopathology or about effective treatments.

5-HT abnormalities may be the result of rather than cause of OCD and OCRD symptoms. Additionally, changes in serotonergic transmission may have direct or indirect effects on the neuronal firing of more than 60 other neuromodulators affecting thoughts, feelings and behaviors. Thus, OCD is probably the final expression of many different kinds of abnormalities in the structure and functioning of the brain. However, this complexity helps us to understand why some treatments are helpful while others are not as effective. The 5-HT hypothesis was initially motivated by the observed differential efficacy of selective SRIs (SSRIs) in alleviating OCD symptoms. These findings, although attesting to the therapeutic versatility of serotonin transporter inhibition in OCD, do not necessarily reflect the existence of neurobiological abnormality in the central serotonergic system in OCD; a reasoning referred to as an *ex juvantibus *argument [37]. There is also growing evidence from both preclinical and clinical studies that the dopamine (DA) system may be involved in the pathogenesis of OCD [38]. Studies on knockout (KO) mice for 5-HT_2C _receptor, already described as a model for obesity, showed increased chewing on non-nutritive clay with a distinct 'neat' pattern and a reduced habituation of head dipping activity as compared to the wild type, with the conclusion that the 5-HT_2C _receptor null mutant mouse provides a putative model for compulsive behavior [39]. Tsaltas *et al*. have described a model based on persistence in the context of rewarded spatial alternation [40]. Using this behavior model, they have shown that 5-HT_2C _receptors are implicated in the mechanisms underlying the 'compulsive' behavior in this animal model for OCD. Acute administration of meta-chlorophenylpiperazine (mCPP), a non-selective 5-HT receptor agonist mainly acting at the 5-HT_2C _receptors but with some affinity also for the 5-HT_1B_, 5-HT_1A _and α_2_-adrenergic receptors, increased 'compulsive' behavior [37]. The selective 5-HT_1B _receptor agonist naratriptan was not effective in this animal model, supporting the role of 5-HT_2C _receptors underlying the effect of mCPP [40]. On the basis of electrophysiological data, Joel and Doljansky suggested that compulsive lever-pressing depends on a phasic decrease in stimulation of D_1 _receptor [41]. In a pharmacological animal model for OCD, in which rats are chronically treated with the selective D_2_/D_3 _receptor agonist quinpirole (QNP), a ritual-like set of behavioral acts resembling OCD checking behavior, has been observed [42]. This 'compulsive' behavior depends on QNP administration, because it rapidly returns to normal behavior when QNP administration is discontinued [43]. Postmortem analysis in these animals revealed increased DA tissue levels in the NAc and right-PFC. The DL-PFC enables temporal information processing and holding relevant information online [44]. It is believed to mediate working memory and executive functions. The basal ganglia are thought to project back to these cortical areas though the medial dorsal and anterior nuclei of the thalamus [45].

A great amount of literature evidence reports OCD and OCRD to show an inherited transmission. Some families have at least four successive generations with clear OCD cases [46]. Since family members could have 'learned' these behaviors from other relatives, the presence of OCD across generations alone is not sufficient to unequivocally prove inheritance [47]. However, successive family members often have different obsessions and compulsions, suggesting that they have not 'learned' them. What appears to be inherited is the capacity to respond to common life experiences with obsessions and compulsions. There are also studies of inheritance involving identical (homozygotic) and fraternal (heterozygotic) twins, which also provide supportive evidence for an inherited component in OCD and OCRDs [48].

Molecular genetics studies have begun to provide evidence that specific genes may play a role in the manifestations of OCD. Segregation analysis has examined familiar patterns of OCD transmission [49,50].

The genetic hypothesis suggests at least few major genes implicated in OCD and OCRDs, thus they are considered oligogenic disorders. Among others, the following regions have been suggested as susceptibility loci: 1q, 6q, 9p, 19q, 7p and 15q. Specifically, the chromosome 11p15 has been linked and associated with a supposed gender effect in the OCD etiology [51]. Interestingly, deletions and other abnormalities of human chromosome 15q11-q13 are associated with two developmental disorders, PWS and Angelman syndrome (AS), which may also present with psychiatric symptoms such as binging and other ICD-related and OCRD-related manifestations [52,53]. Detailed analysis of the 15q11-q14 sequence corrected errors and gaps in the public access sequence to fully reveal large segmental duplications at breakpoints for PWS, AS, and inv dup(15) syndromes, confirming the tight correlations between those conditions [54].

Dhossche *et al*. suggested an association between autism, PWS, AS, catatonia and GABA dysfunction, and this appears to be particularly interesting considering the clinical and partial phenomenic overlap between those conditions, which indeed may appear with OCD and OCRD-related symptoms too [55]. PWS may present with atypical psychotic features and motor dysfunctions characterized by ritualistic, stereotyped and compulsive behaviors, which may be treated with GABA mimetic compounds such as lorazepam, valproic acid and possibly topiramate [56]. Many of the known genetically-based neurodevelopmental disorders are associated with distinctive behavior phenotypes such as this; behavioral phenotypes have been elucidated by clinical research, from distinctive profile or social traits, or have emerged as prominent syndromic features. Social phenotypic findings exist for fragile X syndrome, Down syndrome and PWS, Smith-Magenis syndrome, Turner syndrome, Williams syndrome and velocardiofacial syndrome, all possibly associated with autism [57].

OCD candidate genes have been studied based on their function and also their position in the genome. Serotonin-related genes in OCD include those coding for the 5-HT transporter (5-HT_T_) and receptors (5-HT_2A_, 5-HT_2B_, 5-HT_2C _and 5-HT_1B_) as well the 5-HT enzyme tryptophan hydroxylase [58].

DA-related genes supposed to be implicated in OCD include DA transporter (DA_T_) genes and the D_2_, D_3 _and D_4 _receptors [59,60], as well as the catechol-*O*-methyltransferase (COMT) and monoamine oxidase A (MAO-A) enzymes [61]. Glutamate-related genes (GRIK and GRIN_2B_) and transporters (SLC1A1) have also been investigated in OCD along with the neurotrophic tyrosine kinase type 3 (NTRK3) and other genes such as the white matter genes OLIG_2 _and MOG [62].

However, given the complexity of OCD phenotype, it is unlikely that a single candidate gene will have a major impact on the disorder. Additionally, many individuals suffering from OCD have no family members presenting obsessive-compulsive symptoms.

## Treatment and management

The past 20 years have seen the emergency and evaluation of two major effective forms of treatment for OCD and OCRDs: CBT and drug therapy. Additionally, many other modalities, including physical treatments as electroconvulsive therapy (ECT) and ablative neurosurgical procedures have been proposed as well and should not bet disregarded even today [63].

Drug treatment using medications with marked effect on serotonergic neurotransmission has been shown to be effective in decreasing both obsessions and compulsions, while combining this pharmacological approach with CBT has been reported as the most effective strategy for most OCD and OCRDs cases [64].

Yet, regardless the adopted therapeutic strategy, results vary depending on many factors including the age of onset of the disorder, how long it has been left untreated, the OCRD subtype and/or comorbidity, the patient's insight and compliance and others [8]. Additionally, the therapeutic strategy should be 'tailored' for each single case.

CBT helps patients learn how to quell the discomfort arising from obsessions and how to reduce or eliminate compulsive rituals and it includes exposure and response prevention (ERP) and also cognitive therapy (CT).

Behavior therapy is not something done to a patient: it is a structured set of techniques the patient learns to employ whenever anxiety, discomfort, or dysfunction arise because of obsessions or rituals. Basically, patients are asked to find and face the things they fear ('exposure') and then to refrain from carrying out compulsive rituals ('rituals or response prevention'), but other techniques may be employed as well [65]. Various degrees of success ratio for CBT as OCD or OCRD monotherapy have been reported depending on many factors: the source and method of the study, the session frequency, the OCRD subtype and others [10,66]. Also, CBT has been hypothesized to be associated with brain glucose metabolism improvements for OCD patients [67].

Since the combination of CBT and SRI drugs seems to achieve the best results in clinical settings, this has been proposed as first-line approach in most of cases [64].

Effective SRI treatments for OCD include SSRIs and tricyclic antidepressants (TCAs), especially clomipramine, a tertiary amine. Relatively weak serotonergic TCAs, such as the predominantly norepinephrergic secondary amines, do not tend to be as effective in OCD and OCRD treatment [64]. SRIs also include the serotonergic-norepinephrergic reuptake inhibitors (SNRIs), the serotonin antagonist-reuptake inhibitors (SARIs) and others, but stronger evidence is needed to support their use as effective monotherapy for OCD and OCRDs or, as for the anti-MAOs, relevant clinical side effects may discourage or limit their use [68].

The decision to initiate treatment with SSRI alone, CBT only or a combination, depends on individual patients variables. Non-drug compliance, pregnancy, breastfeeding, very young or very old or mild OCD patients may prefer CBT alone. SSRI treatment should represent the desirable approach in most of the drug-treated cases because of the side effects associated with the TCA clomipramine.

Clomipramine still represents an effective treatment for severe OCD and OCRDs [69], but as the classification of OCD and OCRDs changed with the past editions of DSM so did the therapeutic approaches.

An *ex adiuvantibus *confirmation of partial phenomenic overlap in OCD-related clinical manifestations is historically provided by clomipramine's effectiveness in treating such conditions, also leading researchers focusing on serotonergic mechanism [70]. The pharmacological finding that serotonergic agents are more effective for obsessions and compulsions rather than non-serotonergic antidepressants, and that their anti-obsessive benefit it is independent of the antidepressant action, also contributed to the separation of OCD from mood disorders [71].

When considering the SSRI class, the choice of a specific drug depends on evidence-based medicine but also on the pharmacokinetic and pharmacodynamic properties of the biologically active agent. To mention few, a long-half life (T1/2) should be preferred for very anxious patients, reducing the risk of rebound syndrome (RS) and allowing fewer daily administrations, but it may be not suitable for older patients. Likewise, pharmacodynamic aspects should suggest anticholinergic (anti-Ach), anti-hystaminic type-1 (Anti-H_1_) and/or anti-alpha norepinephrergic type-1 (anti-α_1_) side effects to be preferred when a higher sedation is sought (for example, for very severe ICDs, Tourette's and other OCRDs), while the mild pro-DA agonistic action of others (such as the weak one provided by high-dosage sertraline) should be considered for BDD, binging, craving and other OCRD-related pleasure-seeking behaviors.

Indeed the SSRIs are an almost 5-HT 'selective' class of drugs: weak pharmacodynamic actions could represent a powerful clinical tool when properly managed.

Because many OCD patients respond to treatment with SRIs, usually requiring and tolerating higher doses compared to affective patients, OCD is often deemed a serotonergic dysfunctional disorder. However, despite the 'selective' efficacy of (S)SRIs, many OCD and OCRD patients fail to respond ('non-responders') to adequate doses and time exposures (for example, 20 to 60 mg/day of paroxetine for 12 weeks or 150 to 300 mg/day of clomipramine for 12 weeks), or may require augmentation strategies, usually performed by employing different classes of drugs [64,72]. Additionally, higher doses and the delay in the onset of action can be accounted for by the greater delay in downregulation of serotonergic 5-HT_1B _receptor in the OFC and the subsequent stimulation of 5-HT_2A _receptor antagonists (such as atypical antipsychotics (AA) also known as 5-HT_2A_>D_2 _receptor antagonists) can hasten or augment the effects of SRIs [73]. Consequently, the clinical management of resistant OCD and OCRDs may first consider a hyperdose of SRIs, especially for hard-to-treat forms of OCRD (for example, hoarder-collector patients), prior to augmentation strategies [74]. The augmentation of SSRIs with clomipramine showed significant improvements in Y-BOCS scores compared to SSRI monotherapy, but pharmacokinetic interactions and higher risk for serotonergic malignant syndrome (SMS) may discourage this kind of procedure [75]. Also, SRI anti-obsessive drugs have occasionally been reported to be associated with birth defects, and they should therefore be avoided in pregnant or breastfeeding women (while young or old patients may require lower doses).

Alternative augmentations for resistant OCD and OCRD cases may include the use of low doses of dopaminergic antagonists (haloperidol and pimozide or other typical antipsychotics (TA) or the AA class), especially for poor-insight patients (when compliant). AA may also contribute to mood stabilization in drug-induced manic patients or true bipolars [76]. Also, AAs are less likely to cause extrapyramidal symptoms (EPS) or neuroleptic malignant syndrome (NMS) in sensitive subjects.

The efficacy of adjunctive TAs or AAs to SRIs in refractory OCD may be due to direct dopaminergic D_2 _blockade separate (TA) or together (AA) with 5-HT_2 _receptor antagonism. Additionally, SRI-refractory OCD and OCRDs patients may have additional dysfunctional abnormalities in dopaminergic pathways that may require augmentation with DA-blocking drugs.

**Figure 3:**
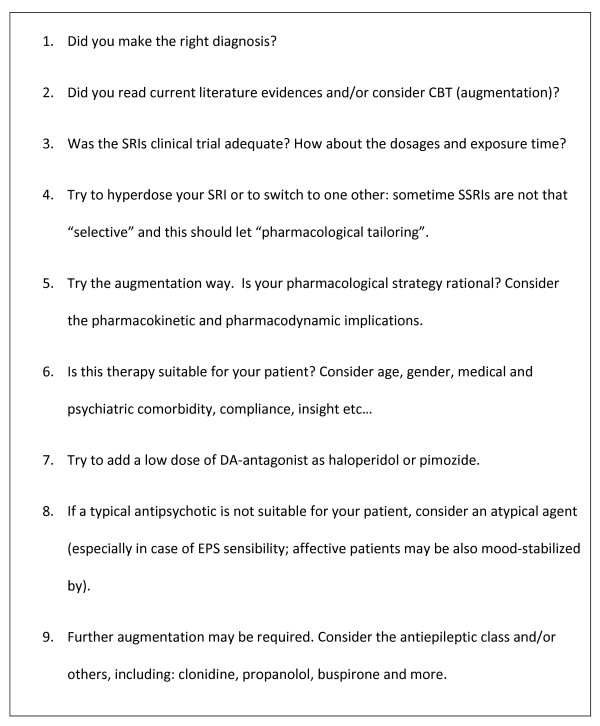
**Drug management of obsessive-compulsive disorder (OCD) and related disorder (OCRD) non-responders**.

Recent neuroimaging findings have also proposed riluzole and other glutamatergic modulators as a possible SRI augmentation strategy for OCD-refractory patients, but further evidence is needed [77-81]. The antiepileptic mood stabilizers such as carbamazepine, topiramate, gabapentin and others, due to the neuroinhibitorial CNS action of GABA in OCD circuits, norepinephrergic α2 (for example, clonidine) and β1 blockers (for example, propanolol), selective 5-HT_1A _partial agonists (buspirone), clonazepam and other benzodiazepines (BDZ), opioid agents (for example, tramadol), antiandrogens and adrenal steroids (for example, flutamide), peptides (for example, oxytocin), hallucinogenetic agents (for example, lysergic acid diethylamide (LDS) and other drugs also acting as 5-HT_2A _partial antagonists), inositol and more, have been proposed, but no univocal opinion on their efficacy for non-responder OCD and OCRDs patients exists (Figure 3).

While a high number of drugs is available for rational OCD therapy, less literature evidence exists about OCRD non-responders management, possibly due to a lower prevalence and clinical recognition of this group; this is why a good pharmacological background should never be missed by the prescriber.

The concept of non-responders also implies a mismatch between a diagnostic classification and treatment, and this may prompt researchers and clinicians to revise current nosography and biological hypothesis [5,82].

## Conclusion

A better understanding of the clinical phenomenology, etiology and therapy of obsessive-compulsive-spectrum disorders will provide clinicians with important information about the effective management of these conditions.

Most OCDs and OCRDs may go underdiagnosed or may be not promptly treated. As a consequence, many patients and their families may suffer unduly, while a delayed therapeutic intervention may mean they do not recover as effectively as an early dagnosis would allow.

Specifically, we suggest the following:

1. An early recognition of OCD and OCRDs will improve outcome and reduce burden.

2. Following current diagnostic criteria may be a useful approach in most cases, but a knowledge of OCD and OCRD phenomenology is a unique opportunity to better address the patient's needs. It also may lead to better compliance between the caregiver and the patient through a deeper understanding of each other goals.

3. To date, no univocal opinion exists about the neurobiological basis of OCD spectrum disorders. Regardless, it is important to know current literature evidence, as this is a core mechanism to proposing a rational therapeutic strategy.

4. Spreading knowledge of OCD and OCRD phenomena may also lead to overcoming the stigma it is associated with, and may finally lead to a greater comprehension of such disorders.

## Competing interests

The authors declare that they have no competing interests.

## Authors' contributions

MF conceived the study and wrote the main document. FG, CA, SF and SR served in the data and reference collection process. CM, PS and VV contributed in document reviewing. PF coordinated data collection and helped to draft the manuscript. All authors read and approved the final manuscript.
